# Clinicopathological features and diagnostic intervals of second primary breast cancer following a prior malignancy: a single-center retrospective study

**DOI:** 10.3389/fonc.2026.1897767

**Published:** 2026-08-17

**Authors:** Yadong Liu, Xuejuan Duan, Jinlong Liu, Peihong Lian, Yibo Li, Yaying Wu, Zixuan He, Xianbo Zhang, Jing Zhao

**Affiliations:** 1 Department of Oncology, Hebei General Hospital, Shijiazhuang, China; 2 Department of Radiation Oncology, The Fourth Affiliated Hospital of Hebei Medical University, Shijiazhuang, China; 3 Graduate School, Hebei North University, Zhangjiakou, China; 4 Hebei Medical University, Shijiazhuang, China

**Keywords:** interval time, molecular typing, multiple primary malignant tumors, prior-breast-cancer subgroup, retrospective study, second primary breast cancer

## Abstract

**Background:**

As cancer survival improves, the number of patients at risk of a second primary malignancy (SPM) is increasing. Previous research has focused mainly on SPMs after breast cancer, whereas clinicopathological data on breast cancer arising after another malignancy remain limited, particularly in Chinese populations.

**Objective:**

To characterize the demographic, clinicopathological, molecular, and temporal features of second primary breast cancer (SPBC) after a prior malignancy and to conduct an exploratory comparison according to whether the first primary cancer was breast cancer.

**Methods:**

A retrospective analysis was performed on 918 patients diagnosed with malignant tumors after prior malignant tumor treatment in Hebei General Hospital from May 2019 to March 2025, among whom 41 patients with SPBC were enrolled. Evaluations were carried out in accordance with the SEER definition of multiple primary tumors, the 8th edition of AJCC TNM staging system and the 2017 St. Gallen consensus on breast cancer molecular typing. Synchronous and metachronous SPBC were defined by intervals of ≤6 months and >6 months, respectively. Statistical analyses were performed using SPSS, and normality was assessed with the Shapiro–Wilk test.

**Results:**

A total of 41 patients were included (40 females and 1 male), with a mean age of 60.3 ± 12.1 years (range, 35–86 years) at SPBC diagnosis. There were 21 cases (51.2%) of left-sided SPBC and 20 cases (48.8%) of right-sided SPBC, and the upper outer quadrant was the most common lesion site accounting for 53.7%. Invasive ductal carcinoma was the predominant pathological type (80.5%). Most cases were in early clinical stages, including stage I (39.0%) and stage II (31.7%). Luminal B was the most prevalent molecular subtype (43.9%), followed by triple-negative breast cancer (22.0%). Breast cancer (namely bilateral breast cancer) was the most common primary tumor (20 cases, 48.8%), followed by gynecological tumors (14.6%, including 5 cases of cervical cancer and 1 case of ovarian cancer), urinary system tumors (12.2%), thoracic tumors (9.8%, all lung cancers) and gastrointestinal tumors (9.8%). The median interval from primary tumor onset to SPBC diagnosis was 60 months (IQR, 46–142 months). Patients with an interval longer than 5 years accounted for 48.8%, those with an interval of 3 to 5 years accounted for 29.3%, and only 2 cases (4.9%) had synchronous tumors with an interval no more than 6 months. The median interval of the prior-breast-cancer subgroup(PBC) was 67 months (IQR, 54.3–153.8 months), and its triple-negative subtype proportion (30.0%) was higher than that of other primary tumor subgroups (14.3%). All patients received surgical treatment for their primary tumors (100%), with chemotherapy exposure rate of 51.2% and radiotherapy exposure rate of 34.1%.

**Conclusion:**

In this selected single-center cohort, 20 of 41 patients had a prior breast cancer, and almost half of SPBC diagnoses occurred more than 5 years after the first malignancy. Luminal B was the most frequent subtype. TNBC was numerically more common in the prior-breast-cancer subgroup, but this difference was not statistically significant. These descriptive findings support risk-adapted long-term surveillance and require validation in larger cohorts with appropriate comparators.

## 1 Introduction

Breast cancer remains one of the most commonly diagnosed malignancies worldwide, with approximately 2.3 million new cases in 2022 (1). In China, national estimates likewise identify breast cancer as the leading cancer by incidence among women (2). Improvements in detection and treatment have expanded the population of breast cancer survivors. Second primary malignancies (SPMs), including contralateral breast and non-breast cancers, are therefore an important concern during long-term follow-up (3, 4).

The development of SPMs is multifactorial and may reflect shared genetic susceptibility, aging, environmental and lifestyle exposures, hormonal factors, treatment effects, and patterns of surveillance (3, 5, 6). Two commonly used frameworks for classifying multiple primary cancers are those of the US Surveillance, Epidemiology, and End Results (SEER) Program and the International Association of Cancer Registries/International Agency for Research on Cancer (IACR/IARC). These frameworks differ in their handling of site, histology, laterality, and timing; in particular, contralateral breast cancer is generally counted as a new primary under SEER rules, whereas the IACR/IARC rules are more conservative (7). This study used the SEER framework and classified contralateral and metachronous ipsilateral breast cancers as second primary malignancies after clinicopathological review. We use the conservative term prior-breast-cancer (PBC) subgroup for patients whose first malignancy was breast cancer.

Most contemporary studies have evaluated cancers arising after a first breast cancer rather than breast cancer occurring after another primary malignancy (3, 4, 8, 9). For example, Oprean et al. (9) reported SPMs in 3.8% of 721 postmenopausal breast cancer patients in western Romania, with contralateral breast, gynecological, and gastrointestinal cancers among the common sites. By contrast, Studies focused on SPBC remain limited, particularly in mainland China. Describing SPBC may clarify its clinicopathological profile, the contribution of patients with a prior breast cancer, and organ-specific diagnostic intervals.

We screened 918 institutional records describing a malignant tumor after treatment of a prior malignancy. Forty-one patients had a later breast malignancy documented as a new primary and met the analytic eligibility criteria. We described the first-cancer distribution, diagnostic intervals, and clinicopathological features of SPBC. Comparisons between the PBC and non-breast first-primary subgroups were exploratory.

## 2 Materials and methods

### 2.1 Study design and participants

**Figure 1:**
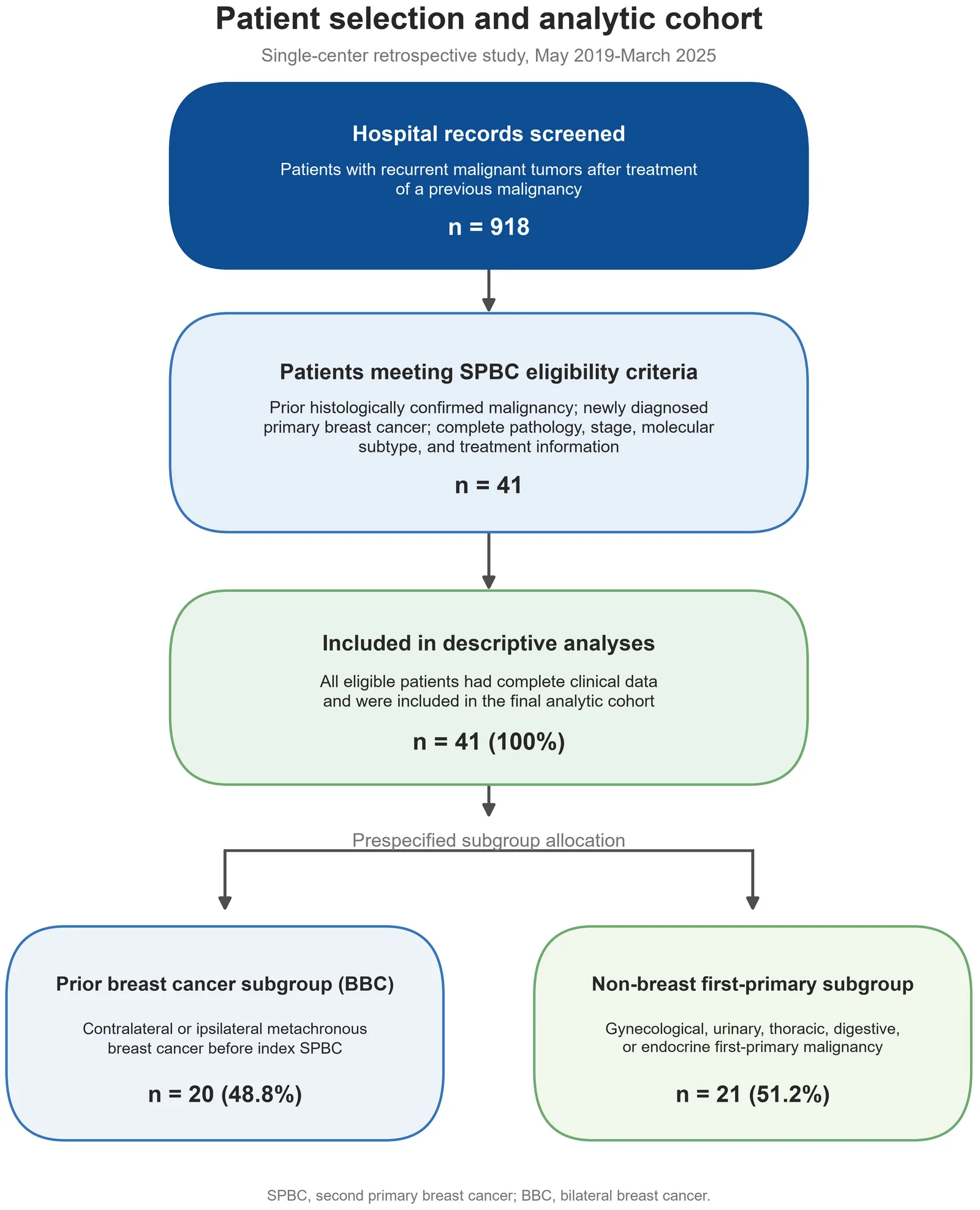
STROBE flow diagram. Patient selection and analytic cohort. Institutional records were screened to identify patients with a later breast malignancy documented as a new primary. The final descriptive cohort comprised 41 patients, including 20 with a prior breast cancer and 21 with a non-breast first primary.

This is a single-center retrospective observational study. The research subjects were patients treated at Hebei General Hospital from May 2019 to March 2025 who met the following inclusion criteria: (1) having a history of any histologically confirmed malignant tumor; (2) subsequently diagnosed with newly-onset primary breast cancer; (3) complete key information including pathological results, staging, molecular typing and treatment regimens. (4) Consistent with the SEER definition of multiple primary tumors. Exclusion criteria: (1) newly detected breast lesions confirmed as distant metastasis rather than primary tumors; (2) patients with severely missing clinical data. A total of 41 patients were finally enrolled. The clinical data of the 41 screened patients were complete; all data were extracted from the electronic medical record system without missing values (**Figure 1**).

### 2.2 Definition of multiple primary tumors and molecular typing

The Surveillance, Epidemiology, and End Results (SEER) program criteria were adopted to define multiple primary tumors: (1) different ICD-O-3 histological codes; (2) an interval of more than 60 days between the diagnosis of carcinoma *in situ* and invasive tumors; (3) different topography codes; (4) tumors arising in the same organ system with a diagnostic interval longer than one year (5). Contralateral breast cancer was defined as the second primary cancer in accordance with SEER rules in this study. A 6-month interval was set as the cutoff to distinguish synchronous and metachronous cancers, namely cancers diagnosed within an interval of no more than 6 months were defined as synchronous cancers.

Breast cancer stage was assigned using the eighth edition of the American Joint Committee on Cancer (AJCC) TNM staging system, according to the year of diagnosis. Molecular subtype was assigned using the clinicopathological surrogate framework applied in the study period, based on ER, PR, HER2, and Ki-67, with reference to the St. Gallen consensus (10). Invasive cancers were categorized as Luminal A, Luminal B, HER2-positive/HR-positive, HER2-positive/HR-negative, or triple-negative. ER or PR expression of ≥1% was considered positive. Because Ki-67 thresholds have varied across consensus eras and laboratories, the >14% cut-off used in this retrospective dataset should be interpreted as a study-specific historical criterion rather than a universally current threshold.

### 2.3 Data collection

We collected age and sex at SPBC diagnosis; SPBC laterality, quadrant, histology, stage, ER, PR, HER2, Ki-67, and surrogate subtype; first-cancer type and treatment exposures; and the interval from the first cancer to SPBC. For the PBC subgroup, the dataset also included selected first-breast histology, HER2, Ki-67, and treatment variables. It did not retain first-breast laterality or standardized paired clonality evidence.

### 2.4 Statistical analysis

IBM SPSS Statistics 22.0 software was used for statistical analysis. Categorical variables were presented as frequencies and percentages. The normality of each continuous variable was assessed using the Shapiro–Wilk test. Normally distributed continuous variables were summarized as mean ± standard deviation, whereas non-normally distributed variables were summarized as median (interquartile range [IQR]; range). Fisher’s exact test was used for between-group comparisons of categorical variables. Because the diagnostic interval was non-normally distributed in at least one subgroup, it was compared between the PBC and non-breast first-primary subgroups using the two-sided Mann–Whitney U test. A two-sided P value <0.05 was considered statistically significant. Given the limited sample size and the mainly descriptive nature of this study, multivariable regression analysis was not performed.

### 2.5 Ethical approval

The study was conducted in accordance with the Declaration of Helsinki and approved by the Ethics Committee of Hebei General Hospital (approval no. 2025-339). The ethics committee waived the requirement for written informed consent because of the retrospective design and de-identification of all research data.

## 3 Results

### 3.1 Baseline demographics and SPBC tumor characteristics

A total of 41 patients with SPBC were finally enrolled in the study, including 40 females (97.6%) and 1 male (2.4%). The age at SPBC diagnosis did not significantly deviate from normality (Shapiro–Wilk W = 0.979, P = 0.653); the mean age was 60.3 ± 12.1 years (range, 35–86 years). In terms of age distribution, patients aged 50–59 years (29.3%) and 60–69 years (29.3%) accounted for the majority, while only 2 patients were under 40 years old (4.9%). There were 21 cases of left-sided SPBC (51.2%) and 20 cases of right-sided SPBC (48.8%). The upper outer quadrant was the most common tumor location (53.7%), followed by the lower outer quadrant (22.0%). Invasive ductal carcinoma was the predominant pathological type, accounting for 33 cases (80.5%). In terms of clinical staging, there were 16 cases in stage I (39.0%), 13 cases in stage II (31.7%), 9 cases in stage III (22.0%) and 3 cases in stage IV (7.3%). As for molecular subtypes, Luminal B subtype accounted for 43.9% (18/41), triple-negative subtype for 22.0% (9/41), Luminal A subtype for 19.5% (8/41), HER2-positive Luminal subtype for 12.2% (5/41), and HER2-positive non-Luminal subtype for 2.4% (1/41). TNBC occurred in 6/20 patients in the prior-breast-cancer subgroup (30.0%; Wilson 95% CI, 14.5%-51.9%) and 3/21 in the non-breast subgroup (14.3%; 95% CI, 5.0%-34.6%). The difference was not significant (Fisher’s exact P = 0.277). Detailed baseline characteristics are shown in **Table 1**.

**Table 1:** Baseline demographic, clinicopathological, and molecular features of 41 patients with second primary breast cancer (SPBC).

Characteristic	No. of patients (N = 41)	%
Age at SPBC diagnosis (years)		
Mean ± SD (range)	60.3 ± 12.1 (35–86)	—
Shapiro–Wilk normality test	W = 0.979; P = 0.653	—
Age group		
<40	2	4.9
40–49	6	14.6
50–59	12	29.3
60–69	12	29.3
70–79	8	19.5
≥80	1	2.4
Sex		
Female	40	97.6
Male	1	2.4
Laterality of SPBC		
Left	21	51.2
Right	20	48.8
Tumor quadrant		
Upper outer	22	53.7
Lower outer	9	22.0
Lower inner	5	12.2
Upper inner	4	9.8
Central	1	2.4
Histology		
Invasive ductal carcinoma	33	80.5
Invasive lobular carcinoma	2	4.9
Ductal carcinoma in situ	3	7.3
Papillary carcinoma	2	4.9
Medullary carcinoma	1	2.4
TNM stage (AJCC 8th)		
I	16	39.0
II	13	31.7
III	9	22.0
IV	3	7.3
Molecular subtype (St. Gallen 2017)		
Luminal A	8	19.5
Luminal B	18	43.9
HER2-positive/HR-positive	5	12.2
HER2-positive/HR-negative	1	2.4
Triple-negative	9	22.0

SPBC, second primary breast cancer; AJCC, American Joint Committee on Cancer; HR, hormone receptor; HER2, human epidermal growth factor receptor 2. Hormone-receptor positivity was defined as ER and/or PR ≥1%; Ki-67 >14% was used as the Luminal A/B cut-off. Ductal carcinoma in situ, Including one case of intraductal papilloma of the breast with low-grade ductal carcinoma in situ within papillary lesions.

### 3.2 Site and system distribution of previous primary malignant tumors

**Figure 2:**
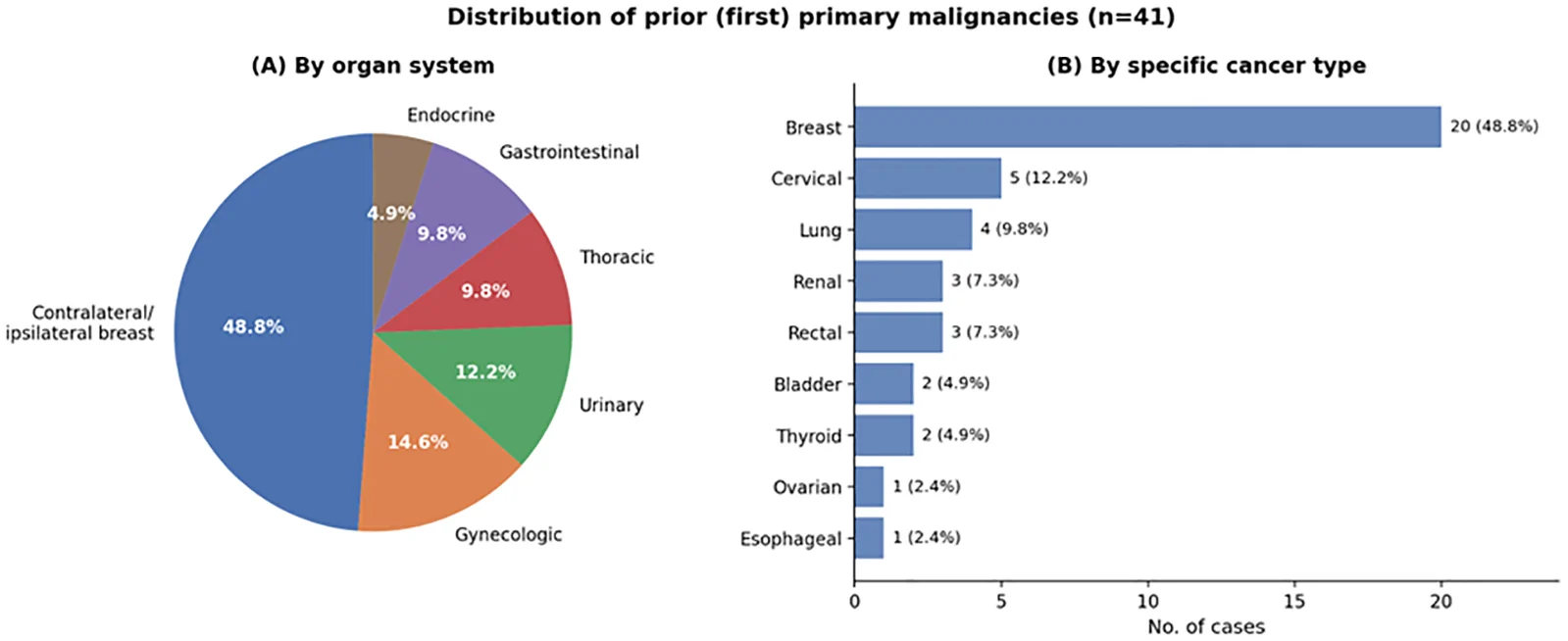
Distribution of the first primary malignancy preceding SPBC (N = 41). **(A)** Distribution by organ system. **(B)** Distribution by specific cancer type. Prior breast cancer was the most frequent first malignancy in this selected cohort.

Among the primary malignant tumors of the 41 patients, breast cancer was the most common, with 20 cases (48.8%), indicating that SPBC was actually metachronous contralateral or ipsilateral breast cancer in survivors with a history of breast cancer. The subsequent common tumor types included 5 cases of cervical cancer (12.2%), 4 cases of lung cancer (9.8%), 3 cases of renal cell carcinoma (7.3%), 3 cases of rectal cancer (7.3%), 2 cases of bladder cancer (4.9%), 2 cases of thyroid cancer (4.9%), and 1 case each of ovarian cancer and esophageal cancer (2.4%). Classified by organ systems, contralateral/ipsilateral breast tumors accounted for 48.8% (20/41), gynecological system tumors 14.6% (6/41, including cervical and ovarian tumors), urinary system tumors 12.2% (5/41, including renal and bladder tumors), thoracic tumors 9.8% (4/41, all lung cancers), digestive tract tumors 9.8% (4/41, including rectal and esophageal tumors), and endocrine system (thyroid) tumors 4.9% (2/41). Relevant data are presented in **Table 2** and **Figure 2**.

**Table 2:** Distribution of the first primary malignancy preceding SPBC, by organ system and specific cancer type (N = 41).

First primary cancer	No. (N = 41)	%
By organ system		
Breast (contralateral/ipsilateral)	20	48.8
Gynecologic (cervical, ovarian)	6	14.6
Genitourinary (renal, bladder)	5	12.2
Thoracic (lung)	4	9.8
Gastrointestinal (rectal, esophageal)	4	9.8
Endocrine (thyroid)	2	4.9
By specific cancer type		
Breast	20	48.8
Cervical	5	12.2
Lung	4	9.8
Renal	3	7.3
Rectal	3	7.3
Bladder	2	4.9
Thyroid	2	4.9
Ovarian	1	2.4
Esophageal	1	2.4

Multiple sites are grouped per SEER organ-system classification; “Breast” denotes a contralateral or ipsilateral metachronous breast cancer occurring before the index SPBC.

### 3.3 Diagnostic interval from primary cancer to SPBC

**Figure 3:**
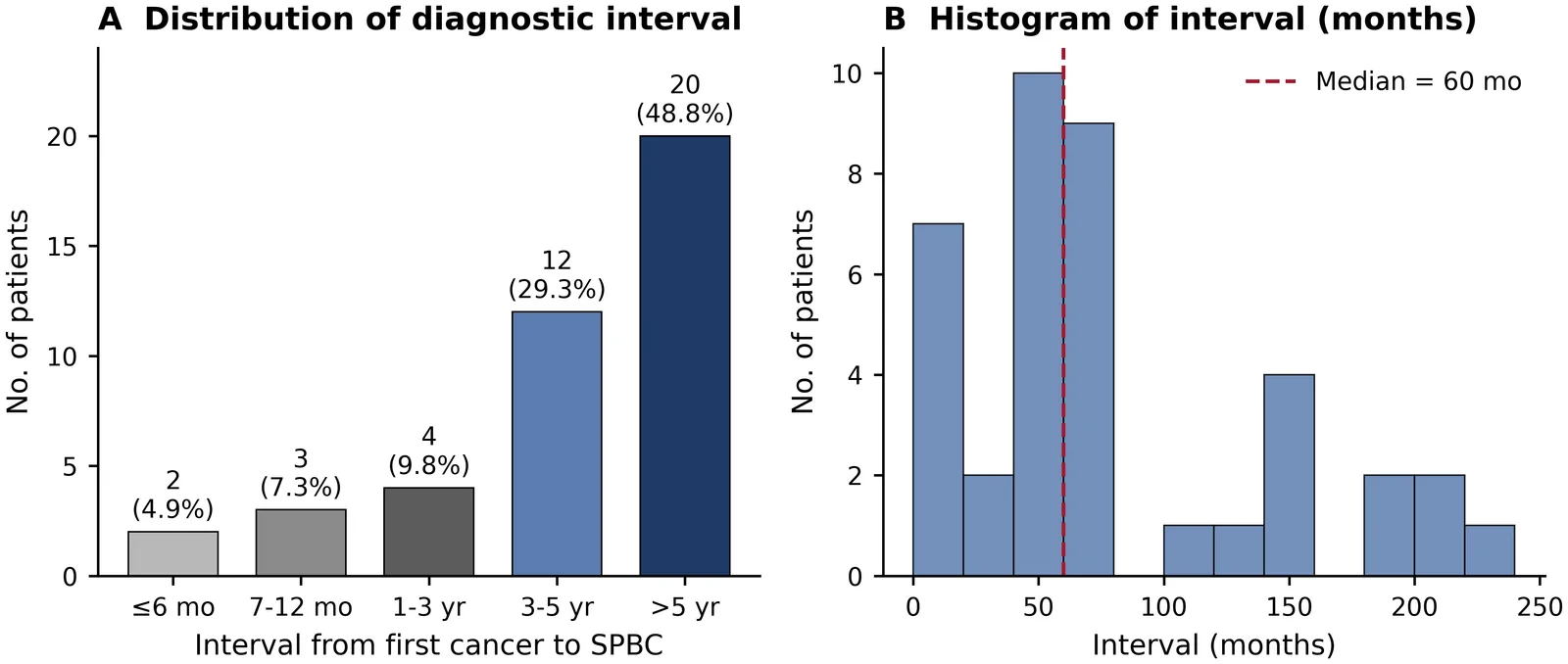
Distribution of the diagnostic interval from the first primary cancer to SPBC (N = 41). **(A)** Categorical distribution of the interval; 48.8% of SPBC events occurred more than 5 years after the first cancer. **(B)** Histogram of the interval in months; the median interval was 60 months (red dashed line).

The diagnostic interval was non-normally distributed in the overall cohort (Shapiro–Wilk W = 0.866, P < 0.001) and had a median of 60 months (IQR, 46–142 months; range, 1–246 months). Among all cases, 2 cases (4.9%) were synchronous cases (≤6 months), and 39 cases (95.1%) were metachronous cases (>6 months). Further refined grouping showed 2 cases (4.9%) within 6 months, 3 cases (7.3%) from 7 to 12 months, 4 cases (9.8%) within 1 to 3 years, 12 cases (29.3%) within 3 to 5 years, and 20 cases (48.8%) over 5 years. Nearly half of SPBC cases occurred more than 5 years after the diagnosis of primary cancer, indicating that long-term follow-up (over 5 years) is particularly vital for monitoring new breast lesions. See **Table 3** and **Figure 3** for details. For treatment of SPBC itself, our dataset allows reliable reporting of endocrine therapy (28/41 patients) and anti-HER2 targeted therapy (6/41 patients). A total of 13 patients received radiotherapy and 25 received chemotherapy.

**Table 3:** Distribution of the diagnostic interval from the first primary cancer to SPBC (N = 41).

Interval from 1st cancer to SPBC	No. (N = 41)	%
≤6 months (synchronous)	2	4.9
7–12 months	3	7.3
>1 to 3 years	4	9.8
>3 to 5 years	12	29.3
>5 years	20	48.8
Median (IQR; range)	(46–142; 1–246) months	—
Shapiro–Wilk normality test	W = 0.866; P < 0.001	—

Synchronous defined as interval ≤6 months from first-cancer to SPBC diagnosis. Approximately one half of SPBCs (48.8%) occurred more than 5 years after the first cancer.

### 3.4 Correlation between primary cancer system and diagnostic interval

**Figure 4:**
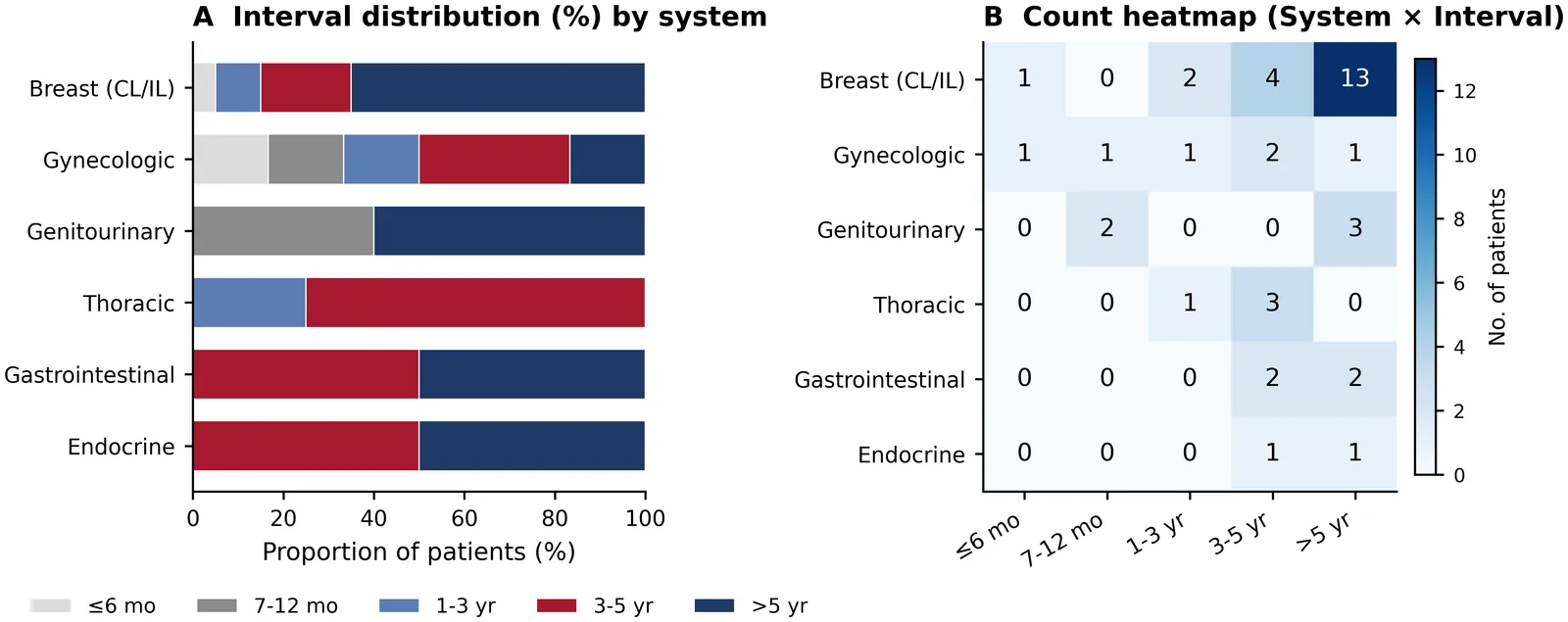
Interval to SPBC stratified by organ system of the first primary cancer (N = 41). **(A)** Stacked proportional bars showing the interval distribution within each first-cancer organ system. **(B)** Count heatmap of the same data. The breast (contralateral/ipsilateral) group was dominated by long intervals (>5 years).

The distribution of intervals stratified by primary cancer systems is presented in **Table 4** and **Figure 4**. The contralateral/ipsilateral breast group had the longest interval, with 13 out of 20 cases (65.0%) occurring over 5 years, suggesting that PBC are mostly metachronous and usually develop more than 5 years after the follow-up of initial breast cancer. The gynecological system group showed the most scattered interval distribution, with 2 out of 6 cases developing within 1 year. All thoracic system cases (all lung cancers) were mostly diagnosed within 3 to 5 years (3 out of 4 cases). Two cases in the urinary system group occurred within 1 year, implying possible synchronous detection via screening. Cases in the digestive and endocrine system groups generally had relatively long intervals. Overall, significant heterogeneity existed in the intervals from primary cancer to SPBC across different organ systems, which needs further verification using survival analysis methods such as cumulative incidence curves in larger sample cohorts.

**Table 4:** Interval from first primary cancer to SPBC, stratified by organ system of the first cancer (N = 41).

First-cancer system	≤6 m	7–12 m	1–3 yr	3–5 yr	>5 yr	Total
Breast (CL/IL)	1	0	2	4	13	20
Gynecologic	1	1	1	2	1	6
Genitourinary	0	2	0	0	3	5
Thoracic	0	0	1	3	0	4
Gastrointestinal	0	0	0	2	2	4
Endocrine	0	0	0	1	1	2
**Total**	**2**	**3**	**4**	**12**	**20**	**41**

Data are number of patients. CL/IL, contralateral/ipsilateral breast cancer. Bold numbers represent the total number of patients in each corresponding subgroup.

### 3.5 Molecular subtypes of SPBC and diagnostic intervals

The distribution of various SPBC molecular subtypes across different interval periods is shown in **Table 5**. Luminal B subtype was the predominant subtype across all interval groups, accounting for 18 cases in total, among which 12 cases (66.7%) were diagnosed over 5 years later. Among 9 triple-negative breast cancer cases, 4 cases (44.4%) occurred more than 5 years after primary cancer diagnosis. All 5 HER2-positive cases were concentrated in the intermediate interval of 1 to 5 years (4/5), with no cases presenting long diagnostic intervals. No typical phenomenon of extremely short intervals (<1 year) accompanied by enrichment of a single subtype was observed in this cohort, indicating that the molecular subtypes of SPBC were relatively evenly distributed across different time windows of onset.

**Table 5:** SPBC molecular subtype across diagnostic-interval categories (N = 41).

SPBC molecular subtype	≤6 m	7–12 m	1–3 yr	3–5 yr	>5 yr	Total
Luminal A	2	1	0	2	3	8
Luminal B	0	2	1	3	12	18
HER2+/HR+	0	0	1	3	1	5
HER2+/HR−	0	0	0	1	0	1
Triple-negative	0	0	2	3	4	9
**Total**	**2**	**3**	**4**	**12**	**20**	**41**

Subtyping per St. Gallen 2017 consensus. Luminal B remained the dominant subtype in all interval strata. Bold numbers represent the total number of patients in each corresponding subgroup.

### 3.6 Primary cancer treatment exposure and SPBC diagnostic intervals

All 41 patients received surgical treatment for their primary cancer (100%). Among them, 21 patients (51.2%) underwent chemotherapy and 14 patients (34.1%) received radiotherapy. Of the 21 patients exposed to chemotherapy, the majority (13/21, 61.9%) developed SPBC more than 5 years after primary cancer confirmation. Among the 14 radiotherapy-exposed patients, 6 cases (42.9%) had SPBC diagnosed over 5 years later, and 4 cases (28.6%) within 3 to 5 years (**Table 6**). Compared with patients without radiotherapy exposure, the proportion of patients developing early-onset SPBC (≤1 year) was similar in radiotherapy-exposed patients, and no obvious correlation between chemotherapy/radiotherapy exposure and early-onset SPBC was identified. This result may be related to limited sample size, insufficient follow-up duration and organ specificity.

**Table 6:** First-cancer treatment exposure and interval to SPBC (treated patients only).

First-cancer treatment	≤1 yr	1–3 yr	3–5 yr	>5 yr	Total
Surgery (n=41)	5 (12.2%)	4 (9.8%)	12 (29.3%)	20 (48.8%)*	41 (100%)
Chemotherapy (n=21)	1 (4.8%)	2 (9.5%)	5 (23.8%)	13 (61.9%)	21 (51.2%)
Radiotherapy (n=14)	1 (7.1%)	3 (21.4%)	4 (28.6%)	6 (42.9%)	14 (34.1%)

Surgery was performed in all 41 patients. *Includes >5 yr category. Values are number (% of treatment-exposed subset).

### 3.7 Exploratory analysis of the prior-breast-cancer subgroup

**Figure 5:**
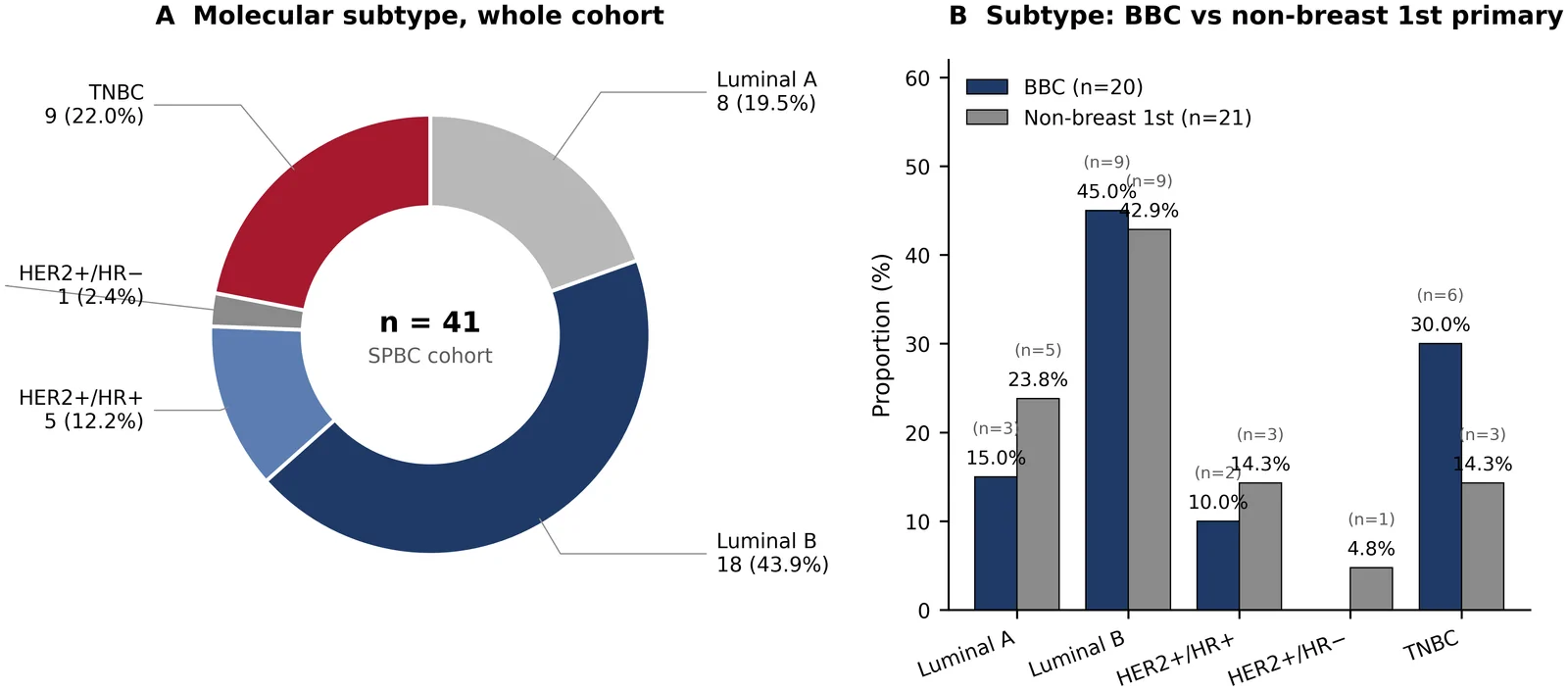
SPBC molecular subtype distribution and subgroup comparison. **(A)** Distribution of SPBC molecular subtypes in the whole cohort (n = 41). **(B)** Comparison of subtype distribution between the Prior-Breast-Cancer Subgroup (n = 20) subgroup and the non-breast first-primary subgroup (n = 21). Triple-negative breast cancer was over-represented in the Prior-Breast-Cancer Subgroup (30.0% vs 14.3%), although the difference did not reach statistical significance.

Twenty patients had a prior breast cancer and formed the PBC subgroup; 21 had a non-breast first primary. The PBC interval median was 67 months (IQR, 54.3-153.8), compared with 49 months (IQR, 22.0-67.0) in the non-breast subgroup (Mann-Whitney U = 285.5, P = 0.0504). Interval-category distributions did not differ significantly (exact P = 0.170). SPBC stage distributions also did not differ (exact P = 0.296). The five-category molecular subtype distribution was similar between groups (exact P = 0.714). TNBC occurred in 6 of 20 PBC patients (30.0%; Wilson 95% CI, 14.5%-51.9%) and 3 of 21 non-breast patients (14.3%; 95% CI, 5.0%-34.6%). This difference was not significant (Fisher’s exact P = 0.277). One PBC patient with TNBC had an explicit BRCA1/2 mutation entry. The remaining entries did not establish whether testing was negative or not performed, so no genetic association was analyzed. Among prior breast cancers, 15 were invasive ductal carcinomas, 1 was HER2-positive, 9 received radiotherapy, 16 received chemotherapy, 5 received endocrine therapy, and none received HER2-targeted therapy (**Table 7**; **Figure 5**).

**Table 7:** Comparison of SPBC clinicopathological features between prior-breast-cancer subgroup and non-breast first-primary subgroups.

Characteristic	Prior-Breast-Cancer Subgroup (n=20)	Non-breast 1st (n=21)	P value†
SPBC stage			
I	6 (30.0%)	10 (47.6%)	
II	6 (30.0%)	7 (33.3%)	
III	5 (25.0%)	4 (19.0%)	
IV	3 (15.0%)	0 (0%)	0.296
SPBC molecular subtype			
Luminal A	3 (15.0%)	5 (23.8%)	
Luminal B	9 (45.0%)	9 (42.9%)	
HER2+/HR+	2 (10.0%)	3 (14.3%)	
HER2+/HR−	0 (0%)	1 (4.8%)	
Triple-negative	6 (30.0%)	3 (14.3%)	0.714
Interval from 1st cancer to SPBC			
Median (IQR), months	67 (54.3–153.8)	49 (22.0–67.0)	0.050
≤6 months	1 (5.0%)	1 (4.8%)	
7–12 months	0 (0%)	3 (14.3%)	
1–3 years	2 (10.0%)	2 (9.5%)	
3–5 years	4 (20.0%)	8 (38.1%)	
>5 years	13 (65.0%)	7 (33.3%)	0.170
First-primary breast cancer features			
Invasive ductal carcinoma	15 (75.0%)	—	—
HER2 positive	1 (5.0%)	—	—
Adjuvant chemotherapy	16 (80.0%)	—	—
Adjuvant radiotherapy	9 (45.0%)	—	—
Endocrine therapy	5 (25.0%)	—	—
HER2-targeted therapy	0 (0%)	—	—

SPBC, second primary breast cancer; HR, hormone receptor; HER2, human epidermal growth factor receptor 2; IQR, interquartile range. †P values were obtained using Fisher’s exact test for categorical variables and the two-sided Mann–Whitney U test for the diagnostic interval; no comparison met the prespecified significance threshold (P < 0.05). “—” indicates not applicable.

## 4 Discussion

This study described the clinicopathological characteristics and diagnostic intervals of SPBC in 41 patients with a previous malignancy. SPBC occurred most often after a first breast cancer, and nearly half of cases were diagnosed more than 5 years after the first malignancy. The predominance of Luminal B tumors and the 22.0% proportion of triple-negative tumors are descriptive findings from this selected cohort and should not be interpreted as population-level enrichment without an appropriate control group. Population-based data show that contralateral breast cancer risk persists over long follow-up, while the magnitude of risk varies with patient, tumor, treatment, and genetic factors (4, 8).

Oprean et al. (9) studied SPMs after breast cancer in 721 postmenopausal women in western Romania; 28 patients (3.8%) developed an SPM, and contralateral breast, gynecological, and gastrointestinal cancers were among the common sites. The present study addressed the reverse clinical perspective—breast cancer arising after any prior malignancy—and found that approximately half of SPBCs were diagnosed more than 5 years after the first cancer. This difference should be interpreted cautiously because of differences in study design, eligibility criteria, age distribution, surveillance, and sample size; it does not establish that SPBC is intrinsically a late-onset disease.

PBC accounted for 48.8% of SPBC cases in this study, a proportion driven by the study’s case-based inclusion criteria and therefore not comparable with the cumulative incidence of contralateral breast cancer in unselected breast cancer populations. In a SEER analysis, the annual contralateral breast cancer risk was approximately 0.37%, with a 25-year actuarial risk of 9.9% (8). Germline BRCA1, BRCA2, and CHEK2 pathogenic variants are associated with increased contralateral breast cancer risk, and risk also varies by age, family history, and tumor characteristics (11, 12). The higher observed proportion of triple-negative disease in the PBC subgroup (30.0% vs 14.3%) was not statistically significant and should be regarded as hypothesis-generating.

Gynecological cancers were the second most frequent group of first malignancies in this cohort. This pattern may reflect shared risk factors and inherited susceptibility, but the small case count does not permit etiological inference. A recent meta-analysis found that breast cancer survivors have increased risks of several non-breast second primaries, including ovarian, uterine, thyroid, kidney, lung, gastrointestinal, and bladder cancers, with variation by population and age at first breast cancer (3). Prospective multiethnic data also support a contribution of germline pathogenic variants in DNA-repair and cancer-predisposition genes to second primary cancer risk (6).

In this small descriptive cohort, SPBC was not observed earlier among patients previously exposed to chemotherapy or radiotherapy. This finding should not be interpreted as evidence for or against treatment causality because treatment allocation, dose, field, latency, competing risks, and baseline susceptibility were not controlled. In a contemporary cohort of 16,004 breast cancer survivors, second-cancer risk varied by treatment type and cancer site, illustrating the need for adequately powered analyses with detailed exposure data (4). For contralateral breast cancer specifically, shared genetic and hormonal susceptibility may coexist with treatment-related effects.

Clinically, the findings support risk-adapted long-term breast surveillance rather than a uniform protocol for all cancer survivors. Annual mammography is recommended for individuals with a personal history of breast cancer and residual breast tissue; supplemental MRI is generally considered for selected higher-risk patients, including some younger patients, those with dense breasts, or those with substantial inherited risk (13). Germline BRCA1/2 testing should be offered to patients who develop a second primary cancer in the ipsilateral or contralateral breast, and broader multigene testing may be appropriate according to personal and family history and its potential to inform management (14). Surveillance and genetic testing decisions should therefore be individualized rather than inferred solely from this cohort’s tumor-site distribution.

### 4.1 Limitations

This study has several limitations. First, it is a small, single-center retrospective case series without a population-based or single-primary breast cancer comparator. Referral, surveillance, and complete-record selection may limit generalizability. Second, the supplied dataset did not retain first-breast laterality, systematic paired histopathological adjudication, standardized paired IHC review, or molecular clonality testing. Misclassification of a later breast lesion as a new primary cannot therefore be completely excluded. Third, the BRCA1/2 field contained one explicit mutation entry, but the remaining values did not distinguish negative tests from unperformed tests. Fourth, radiotherapy field, dose, fractionation, and dates were unavailable, preventing evaluation of thoracic exposure or radiation-induced carcinogenesis. Fifth, survival outcomes were unavailable, and the sample size precluded multivariable modeling. The diagnostic intervals are descriptive and cannot estimate standardized incidence, mortality, or causal risk.

## 5 Conclusions

This single-center case series describes the clinicopathological and temporal features of SPBC after a prior malignancy. Twenty patients had a prior breast cancer, and 20 of 41 SPBC diagnoses occurred more than 5 years after the first malignancy. Luminal B was the most frequent subtype. TNBC was numerically more frequent in the PBC subgroup, but neither the overall subtype distribution nor the TNBC comparison was significant. The findings support further study of risk-adapted long-term surveillance but do not establish subtype enrichment, genetic association, or treatment-related causality.

## Data Availability

Publicly available datasets were analyzed in this study. If you require the raw data, please contact the corresponding author JZ at zzzjmail@163.com.
