# Supplementary material for: Clinicopathological features and diagnostic intervals of second primary breast cancer following a prior malignancy: a single-center retrospective study
Source: Front Oncol. 2026 Aug 17;16:1897767. doi: 10.3389/fonc.2026.1897767 (PMC13526592; doi:10.3389/fonc.2026.1897767)
Supplement: Supplementary file 1 [file DataSheet1.pdf]

STROBE Statement—checklist of items that should be included in reports of observational studies

	Item No	Recommendation	Page(s) / N/A
**Title and abstract**			
	1	(*a*) Indicate the study's design with a commonly used term in the title or the abstract	p. 1
		(*b*) Provide in the abstract an informative and balanced summary of what was done and what was found	pp. 1-2
**Introduction**			
Background/rationale	2	Explain the scientific background and rationale for the investigation being reported	pp. 3-4
Objectives	3	State specific objectives, including any prespecified hypotheses	p. 4
**Methods**			
Study design	4	Present key elements of study design early in the paper	p. 4
Setting	5	Describe the setting, locations, and relevant dates, including periods of recruitment, exposure, follow-up, and data collection	p. 4
Participants	6	(*a*) *Cohort study*—Give the eligibility criteria, and the sources and methods of selection of participants. Describe methods of follow-up<br>*Case-control study*—Give the eligibility criteria, and the sources and methods of case ascertainment and control selection. Give the rationale for the choice of cases and controls<br>*Cross-sectional study*—Give the eligibility criteria, and the sources and methods of selection of participants	pp. 4-5
		(*b*) *Cohort study*—For matched studies, give matching criteria and number of exposed and unexposed<br>*Case-control study*—For matched studies, give matching criteria and the number of controls per case	N/A
Variables	7	Clearly define all outcomes, exposures, predictors, potential confounders, and effect modifiers. Give diagnostic criteria, if applicable	pp. 5-6
Data sources/ measurement	8*	For each variable of interest, give sources of data and details of methods of assessment (measurement). Describe comparability of assessment methods if there is more than one group	pp. 5-7
Bias	9	Describe any efforts to address potential sources of bias	pp. 5-7; 21-22
Study size	10	Explain how the study size was arrived at	pp. 4-5
Quantitative variables	11	Explain how quantitative variables were handled in the analyses. If applicable, describe which groupings were chosen and why	pp. 6-7
Statistical methods	12	(*a*) Describe all statistical methods, including those used to control for confounding	p. 7
		(*b*) Describe any methods used to examine subgroups and interactions	p. 7; pp. 16-18
		(*c*) Explain how missing data were addressed	p. 5
		(*d*) *Cohort study*—If applicable, explain how loss to follow-up was addressed<br>*Case-control study*—If applicable, explain how matching of cases and controls was addressed<br>*Cross-sectional study*—If applicable, describe analytical methods taking account of sampling strategy	N/A

		(*e*) Describe any sensitivity analyses	N/A

**Results**			**Page(s) / N/A**
Participants	13*	(a) Report numbers of individuals at each stage of study—eg numbers potentially eligible, examined for eligibility, confirmed eligible, included in the study, completing follow-up, and analysed	pp. 4-5
		(b) Give reasons for non-participation at each stage	N/A
		(c) Use a flow diagram and include the figure number (preferably figure 1) or page number	Fig. 5
Descriptive data	14*	(a) Give characteristics of study participants (eg demographic, clinical, social) and information on exposures and potential confounders	pp. 8-18
		(b) Indicate number of participants with missing data for each variable of interest	p. 5
		(c) *Cohort study*—Summarise follow-up time (eg, average and total amount)	pp. 11-12
Outcome data	15*	*Cohort study*—Report numbers of outcome events or summary measures over time	N/A
		Case-control study—Report numbers in each exposure category, or summary measures of exposure	N/A
		Cross-sectional study—Report numbers of outcome events or summary measures	pp. 8-18
Main results	16	(*a*) Give unadjusted estimates and, if applicable, confounder-adjusted estimates and their precision (eg, 95% confidence interval). Make clear which confounders were adjusted for and why they were included	N/A
		(*b*) Report category boundaries when continuous variables were categorized	pp. 11-12
		(*c*) If relevant, consider translating estimates of relative risk into absolute risk for a meaningful time period	N/A
Other analyses	17	Report other analyses done—eg analyses of subgroups and interactions, and sensitivity analyses	pp. 15-18
**Discussion**			
Key results	18	Summarise key results with reference to study objectives	pp. 18-21
Limitations	19	Discuss limitations of the study, taking into account sources of potential bias or imprecision. Discuss both direction and magnitude of any potential bias	pp. 19-22
Interpretation	20	Give a cautious overall interpretation of results considering objectives, limitations, multiplicity of analyses, results from similar studies, and other relevant evidence	pp. 18-22
Generalisability	21	Discuss the generalisability (external validity) of the study results	pp. 21-22
**Other information**			
Funding	22	Give the source of funding and the role of the funders for the present study and, if applicable, for the original study on which the present article is based	N/A

*Give information separately for cases and controls in case-control studies and, if applicable, for exposed and unexposed groups in cohort and cross-sectional studies.

*N/A stands for not applicable and may be a reasonable choice depending on the type of study performed
